# Temperature and Development Impacts on Housekeeping Gene Expression in Cowpea Aphid, *Aphis craccivora* (Hemiptera: Aphidiae)

**DOI:** 10.1371/journal.pone.0130593

**Published:** 2015-06-19

**Authors:** Chunxiao Yang, Huipeng Pan, Yong Liu, Xuguo Zhou

**Affiliations:** 1 Hunan Academy of Agricultural Sciences, Institute of Plant Protection, Changsha, Hunan, China; 2 Department of Entomology, University of Kentucky, Lexington, KY, United States of America; Natural Resources Canada, CANADA

## Abstract

Quantitative real-time PCR (qRT-PCR) is a powerful technique to quantify gene expression. To standardize gene expression studies and obtain more accurate qRT-PCR analysis, normalization relative to consistently expressed housekeeping genes (HKGs) is required. In this study, ten candidate HKGs including *elongation factor 1 α* (*EF1A*), *ribosomal protein L11 *(*RPL11*), *ribosomal protein L14 *(*RPL14*), *ribosomal protein S8 *(*RPS8*), *ribosomal protein S23 *(*RPS23*), *NADH-ubiquinone oxidoreductase *(*NADH*), *vacuolar-type H+-ATPase *(*ATPase*), *heat shock protein 70 *(*HSP70*), *18S ribosomal RNA *(*18S*), and *12S ribosomal RNA *(*12S*) from the cowpea aphid, *Aphis craccivora* Koch were selected. Four algorithms, *geNorm*, *Normfinder*, *BestKeeper*, and the *ΔC_t_* method were employed to evaluate the expression profiles of these HKGs as endogenous controls across different developmental stages and temperature regimes. Based on *RefFinder*, which integrates all four analytical algorithms to compare and rank the candidate HKGs, *RPS8*, *RPL14*, and *RPL11* were the three most stable HKGs across different developmental stages and temperature conditions. This study is the first step to establish a standardized qRT-PCR analysis in *A*. *craccivora* following the MIQE guideline. Results from this study lay a foundation for the genomics and functional genomics research in this sap-sucking insect pest with substantial economic impact.

## Introduction

Quantitative real-time PCR (qRT-PCR) is a powerful technique to quantify gene expressions during different biological processes [1]. Although qRT-PCR is one of the premier research tools, limitations still exist, several factors can influence the threshold cycle values including RNA quality, cDNA concentration, and PCR efficiency [2,3]. The most extensively adopted approach in qRT-PCR analysis is to normalize the expressions of target genes through measuring in parallel the expression of a housekeeping gene (HKG). Housekeeping genes, involved in basic cellular functions, are typically believed to possess inherent stable and constitutive expression in different samples under various biotic and abiotic conditions [1].

The cowpea aphid, *Aphis craccivora* Koch (Hemiptera, Aphidiae), is an important pest of cowpea, *Vigna unguiculata* (L.), one of the most important food crops in the semiarid tropical regions, including Asia, Africa, southern Europe, and Central and South America. *Aphis craccivora* typically feeds on several species of legumes (family *Fabaceae*) worldwide, including alfalfa, beans, chickpea, lentils, lupins, and peanuts. Aphids can infest cowpea through direct feeding on leaves, pods and other aerial tissues of the plant, or indirectly through the transmission of virus diseases [4–6]. *A*. *craccivora* can cause great damage even at low population densities because of its ability to transmit at least 14 viruses including the potyviruses, the cowpea aphid-borne mosaic virus and the blackeye cowpea mosaic virus [6,7]. In order to better understanding the molecular basis and facilitate the development of integrated pest management strategies of *A*. *craccivora*, Roche 454 pyrosequencing technology was used to generate the transcriptome of *A*. *craccivora* [7]. To take advantage of these genomics resources, establishing a standardized qRT-PCR procedure in *A*. *craccivora* following the MIQE (Minimum Information for publication of Quantitative real time PCR Experiments) guidelines [8] will be instrumental for the subsequent functional and epi-genomic research.

The objective of this research was to address an important aspect of gene expression studies in *A*. *craccivora*. Here, ten candidate HKGs including *elongation factor 1 α* (*EF1A*), *ribosomal protein L11* (*RPL11*), *ribosomal protein L14* (*RPL14*), *ribosomal protein S8* (*RPS8*), *ribosomal protein S23* (*RPS23*), *NADH-ubiquinone oxidoreductase* (*NADH*), *vacuolar-type H+-ATPase* (*ATPase*), *heat shock protein 70* (*HSP70*), *18S ribosomal RNA* (*18S*), and *12S ribosomal RNA* (*12S*) were selected from the publically available *A*. *craccivora* transcriptome resources and the sequence obtained from GenBank [7]. The expression profile of these candidate HKGs was investigated across different developmental stages and under various temperature regimes. As a result, a suite of reference genes were recommended for the qRT-PCR analysis in *A*. *craccivora*.

## Materials and Methods

### Ethics statement

The cowpea aphid, *Aphis craccivora* Koch (Hemiptera, Aphidiae), was collected from a greenhouse on fava bean, *Vicia faba* (Fabales, Fabaceae), at the University of Kentucky. *Aphis craccivora* colony was maintained on seedlings of fava bean in a growth chamber at 23°C with a photoperiod of 12: 12 (L: D) and 50% relative humidity. No specific permit was required for the described collection. *A*. *craccivora* is a common aphid species with agricultural importance in the United States.

### Samples preparation

For the developmental stage treatment, 10 *A*. *craccivora* adults (only unwinged individuals) and 20 nymphs (mixed nymphal stages) were, respectively, placed on fava bean leaves resting on a wet filter paper in a petri dish (9 cm diameter) for 2 d at 22°C. There are six replicates for the adult and nymph stages, respectively; therefore, there were 12 biological samples in total. For the temperature treatment, 10 *A*. *craccivora* adults and 20 nymphs (mixed nymphal stages) were, respectively, exposed to 10°C, 22°C, and 30°C, respectively, for 2 d. Each treatment was repeated three times independently, therefore, there were 18 biological samples for the temperature experiment. All the experiments were conducted in a growth chamber with a photoperiod of 14: 10 (L: D) and 50% relative humidity. After treatments, aphids were initially snap frozen in liquid nitrogen in a 1.5 ml microcentrifuge tube and then stored at -80°C for the subsequent total RNA extraction.

### Total RNA extraction and cDNA synthesis

Total RNA was extracted using TRIzol reagent (Invitrogen, Carlsbad, CA) according to previously described methods [9,10]. First strand cDNA was synthesized from 1 μg of total RNA with M-MLV reverse transcription kit (Invitrogen, USA) using a random N primer according the manufacturer’s recommendations.

### Reference gene selection and primer design and quantitative real-time PCR

A total of 10 housekeeping genes that are commonly used in qRT-PCR analysis were selected as the candidate (Table 1). Majority of these genes have been previously used as the reference genes in other insect species [10–25]. Primers for *EF1A* was designed based on the sequences obtained from GenBank, and the others were obtained from the transcriptome of *A*. *craccivora* [7]. Primers for the qRT-PCR analysis were designed online, https://www.idtdna.com/Primerquest/Home/Index. The information of qRT-PCR amplifications and programs were described in detail in our previous study [9,10]. The standard curve and PCR efficiency of each candidate were constructed and calculated according to previously described methods [9,10].

**Table 1:** Summary of the ten housekeeping genes tested in this study.

Gene	Description	Accession No.	Primer sequences (5’-3’)	Length (bp)	Efficiency (%)	Regression coefficient
*EF1A*	*elongation factor 1 α*	KC897473	F: CCAGTAGGTCGTGTTGAAACT	100	102.6	0.9997
			R: GGTGCATCTCCACGGATTTA			
*NADH*	*NADH-ubiquinone oxidoreductase*	GAJW01000104	F: CCTCAGCCTATTGAACGAGAAG	101	109.6	0.9976
			R: CCTGCCAGTTCCAGTACTAATC			
*HSP70*	*70 kilodalton heat shock proteins*	GAJW01000112	F: AGTACCATGGAACCCGTAGA	91	99.7	0.9992
			R: GGGTAGAACCTCCAACCAATAC			
*18S*	*18S ribosomal RNA*	GAJW01000254	CCTACCGTCGACAGTTGATAAG	100	95.8	0.9992
			CAAAGACCTGGTGACTCTGAATA			
*12S*	12S ribosomal RNA	GAJW01000011	AGAAACCAACCTGGCTTACAC	121	102.3	0.9992
			TTGCGACCTCGATGTTGAATTA			
*RPS23*	*ribosomal protein S23*	GAJW01000179	TACTGCCCGTAAACACGTAAA	110	95.5	0.9983
			AAGCTCCTCCGAAAGGATTG			
*RPS8*	*ribosomal protein S8*	GAJW01000269	GTCGTCCGAGCCATTCTTT	105	94.8	0.9977
			TCCTGTCTTCCTGCGTTTATG			
*RPL14*	*ribosomal protein L14*	GAJW01000046	CGAGTGGTCTACGTTGTTGAT	106	93.9	0.9993
			GTACTCCAGTTTCTGGTCCATC			
*RPL11*	*ribosomal protein L11*	GAJW01000099	GGAACCACTTCATTGCATCTTC	104	106.3	0.9991
			TGTCTTAGGACGTCAAGGTTTC			
*ATPase*	*vacuolar type H^+^-ATPase*	GAJW01000023	AGAGTGTCCACCATAGTTAGTTG	95	101.3	0.9951
			ATCTCGGTAGTGGGTAGTTAGA			

### Stability of gene expression

All biological replicates were used to calculate the average *C*_*t*_ value. The stability of the ten HKGs was evaluated by algorithms *geNorm* [1], *NormFinder* [26], *BestKeeper* [27], and the comparative *ΔC*_*t*_ method [28]. Finally, we compared and ranked the tested candidate HKGs based on a web-based analysis tool *RefFinder* (http://www.leonxie.com/referencegene.php).

## Results

### Transcriptional profiling of candidate reference genes

The entire candidate HKGs were visualized as a single amplicon with expected size on a 1.5% agarose gel (S1 Fig). Furthermore, gene-specific amplification was confirmed by a single peak in real-time melting-curve analysis (S2 Fig). Standard curves were created for all the candidate HKGs, and the PCR efficiency and correlation coefficient for each standard curve were shown in Table 1.

**Fig 1:**
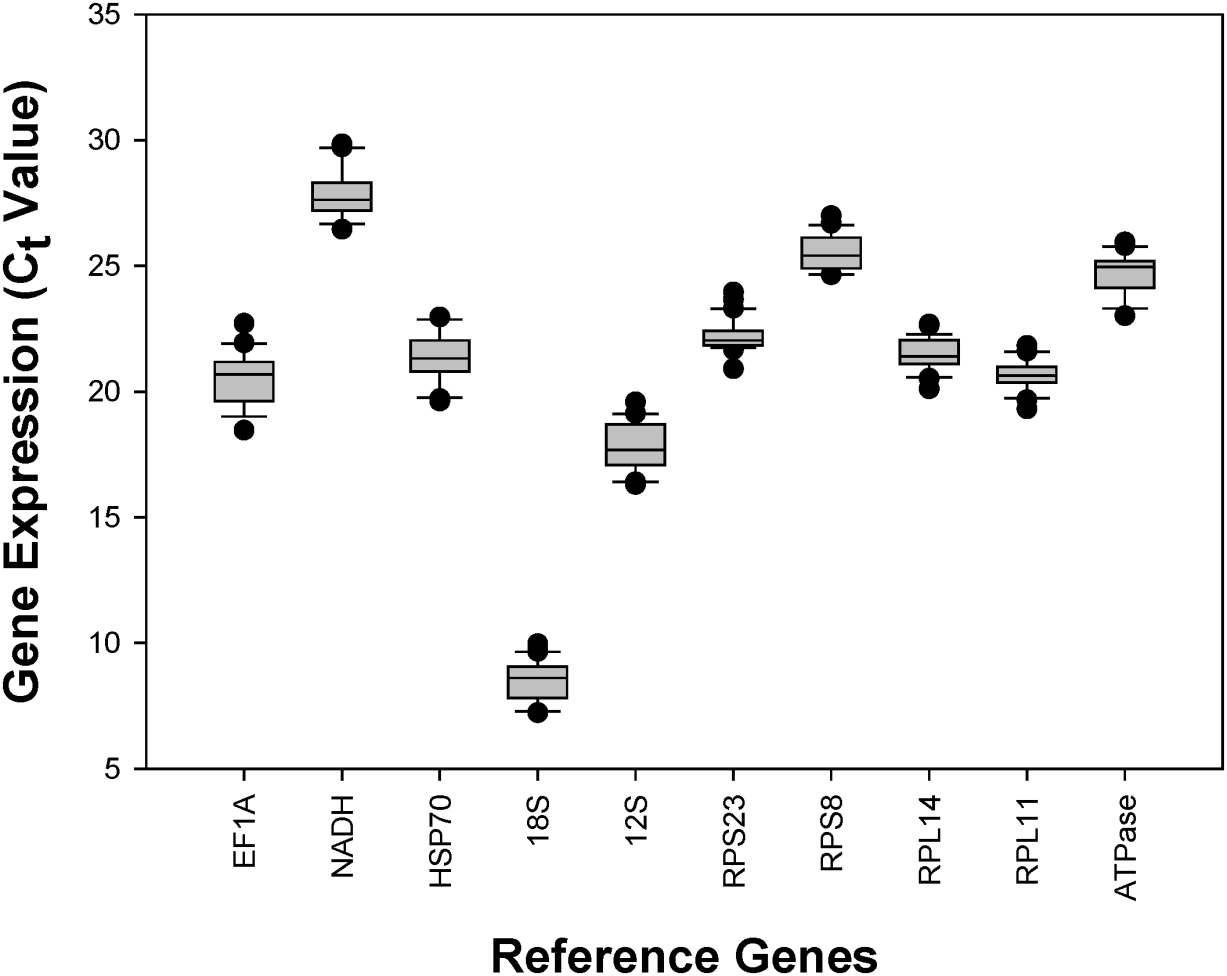
Expression profiles of candidate housekeeping genes in *Aphis craccivora*. The expression level of candidate housekeeping genes in 30 tested samples are documented in *C*_*t*_ value. The median is represented by the line in the box. The interquartile rang is bordered by the upper and lower edges, which indicate the 75^th^ and 25^th^ percentiles, respectively.

The mean and the standard deviation (SD) of the *C*_*t*_ values were calculated for all the samples (S1 Table). *RPL11* (SD = 0.61) had the least variable expression level and it was reflected in its low SD values. On the contrary, *EF1A* (SD = 1.09) had the most variable expression levels, and it was shown in its high SD values. Additionally, *18S* had the lowest *C*_*t*_ values (*C*_*tavg*_ = 8.50), suggesting that it had the highest expression level, whereas, *NADH* was the least expressed gene among the candidates (*C*_*tavg*_ = 27.82) (Fig 1, S1 Table).

### Selection of the best candidate reference genes

Based on *geNorm*, under the impact of temperature, *RPL14* and *RPS8* were co-ranked as the most stable genes. The overall order from the most stable to the least stable reference genes was: *RPL14* = *RPS8*, *RPL11*, *RPS23*, *ATPase*, *12S*, *HSP70*, *NADH*, *EF1A*, *18S* (Table 2). Under the impact of development, *RPL14* and *RPS8* were co-ranked as the most stable genes. The overall order from the most stable to the least stable reference genes was: *RPL14* = *RPS8*, *RPL11*, *ATPase*, *RPS23*, *12S*, *HSP70*, *NADH*, *EF1A*, *18S* (Table 3).

**Table 2:** A summary of ranking for reference gene candidates using five different statistical approaches.

*RefFinder*		*geNorm*		*NormFider*		*ΔCt*		*BestKeeper*			
Genes	GM	Genes	SV	Genes	SV	Genes	SV	Genes [r]		Genes SD	
*RPS8*	1.19	*RPL14*	1.035	*RPS8*	0.792	*RPS8*	1.45	*RPL14*	0.860	*HSP70*	0.88
*RPL14*	2.00	*RPS8*	1.035	*RPL14*	0.805	*RPL14*	1.46	*RPL11*	0.748	*RPS8*	0.95
*RPL11*	3.00	*RPL11*	1.092	*RPL11*	0.955	*RPL11*	1.54	*18S*	0.700	*RPL11*	0.97
*HSP70*	3.64	*ATPase*	1.167	*ATPase*	1.14	*ATPase*	1.62	*RPS8*	0.670	*RPL14*	1.01
*ATPase*	4.47	*RPS23*	1.227	*HSP70*	1.238	*HSP70*	1.69	*ATPase*	0.376	*ATPase*	1.03
*RPS23*	6.26	*12S*	1.276	*NADH*	1.367	*NADH*	1.77	*EF1A*	0.299	*RPS23*	1.04
*12S*	6.74	*HSP70*	1.432	*12S*	1.388	*12S*	1.78	*HSP70*	0.276	*12S*	1.04
*NADH*	6.93	*NADH*	1.52	*RPS23*	1.394	*RPS23*	1.78	*NADH*	0.231	*NADH*	1.05
*EF1A*	9.24	*EF1A*	1.578	*18S*	1.488	*EF1A*	1.86	*12S*	0.183	*EF1A*	1.26
*18S*	9.74	*18S*	1.639	*EF1A*	1.512	*18S*	1.89	*RPS23*	0.001	*18S*	1.40

12 samples were from developmental stage group as input.

Geometric mean (GM); Stability Value (SV); Pearson’s correlation coefficient ([r]); Standard Deviation (SD).

**Table 3:** A summary of ranking for reference gene candidates using five different statistical approaches.

*RefFinder*		*geNorm*		*NormFider*		*ΔCt*		*BestKeeper*			
Genes	GM	Genes	SV	Genes	SV	Genes	SV	Genes [r]		Genes SD	
*RPS8*	1.41	*RPL14*	0.775	*RPS8*	0.813	*RPS8*	1.44	*RPL11*	0.692	*RPL14*	0.86
*RPL14*	1.73	*RPS8*	0.775	*RPL11*	0.883	*RPL14*	1.48	*RPS8*	0.624	*ATPase*	0.89
*RPL11*	2.45	*RPL11*	0.912	*RPL14*	0.976	*RPL11*	1.51	*RPL14*	0.605	*RPL11*	0.90
*ATPase*	3.36	*RPS23*	1.142	*ATPase*	1.009	*ATPase*	1.52	*EF1A*	0.477	*RPS8*	0.94
*RPS23*	5.23	*ATPase*	1.225	*12S*	1.204	*HSP70*	1.64	*18S*	0.462	*RPS23*	1.00
*12S*	5.96	*12S*	1.284	*RPS23*	1.206	*NADH*	1.65	*HSP70*	0.402	*HSP70*	1.01
*HSP70*	6.74	*HSP70*	1.469	*HSP70*	1.242	*12S*	1.68	*ATPase*	0.375	*12S*	1.04
*NADH*	8.00	*NADH*	1.569	*NADH*	1.364	*RPS23*	1.77	*12S*	0.359	*NADH*	1.14
*EF1A*	9.24	*EF1A*	1.632	*EF1A*	1.439	*EF1A*	1.81	*NADH*	0.249	*18S*	1.16
*18S*	9.74	*18S*	1.684	*18S*	1.522	*18S*	1.88	*RPS23*	0.177	*EF1A*	1.30

18 samples were from temperature group as input.

Geometric mean (GM); Stability Value (SV); Pearson’s correlation coefficient ([r]); Standard Deviation (SD).

According to the *ΔC*_*t*_ method, under the impact of temperature, *RPS8* was the top-ranked gene. The overall order from the most stable to the least stable reference genes was: *RPS8*, *RPL14*, *RPL11*, *ATPase*, *HSP70*, *NADH*, *12S*, *RPS23*, *EF1A*, *18S* (Table 2, S2 Table). Under the impact of development, *RPS8* was also the top-ranked gene. The overall order from the most stable to the least stable reference genes was: *RPS8*, *RPL14*, *RPL11*, *ATPase*, *HSP70*, *NADH*, *12S*, *RPS23*, *EF1A*, *18S* (Table 3, S3 Table).

Based on *NormFinder*, under the impact of temperature, *RPS8* was the most reliable and stable reference gene. The overall order from the most stable to the least stable reference genes was: *RPS8*, *RPL11*, *RPL14*, *ATPase*, *12S*, *RPS23*, *HSP70*, *NADH*, *EF1A*, *18S* (Table 2). Under the impact of development, *RPS8* was also the top-ranked gene. The overall order from the most stable to the least stable reference genes was: *RPS8*, *RPL14*, *RPL11*, *ATPase*, *HSP70*, *NADH*, *12S*, *RPS23*, *18S*, *EF1A* (Table 3).

According to *BestKeeper*, the stability of a gene is directly proportional to the [r] value, while it is inversely proportional to the SD value. Under the impact of temperature, *RPL11* had the highest [r] value, and *RPL14* had the lowest SD value across all the samples (Table 2, S4 Table). Under the impact of development, *RPL14* had the highest [r] value, and *HSP70* had the least variable expression levels across all the samples (Table 3, S5 Table)

### Comprehensive ranking of best reference genes using *RefFinder*

Under the impact of temperature, according to *RefFinder*, the comprehensive ranking of candidate reference genes from the most to the least stable was: *RPS8*, *RPL14*, *RPL11*, *ATPase*, *RPS23*, *12S*, *HSP70*, *NADH*, *EF1A*, *18S* (Table 2). Under the impact of development, the comprehensive ranking of candidate reference genes from the most to the least stable was: *RPS8*, *RPL14*, *RPL11*, *HSP70*, *ATPase*, *RPS23*, *12S*, *NADH*, *EF1A*, *18S* (Table 3). Interestingly, *RPL11*, *RPS8*, and *RPL14* were the three most stable HKGs throughout different developmental stages and temperature conditions.

### Quantitative analysis of candidate reference genes based on *geNorm*

**Fig 2:**
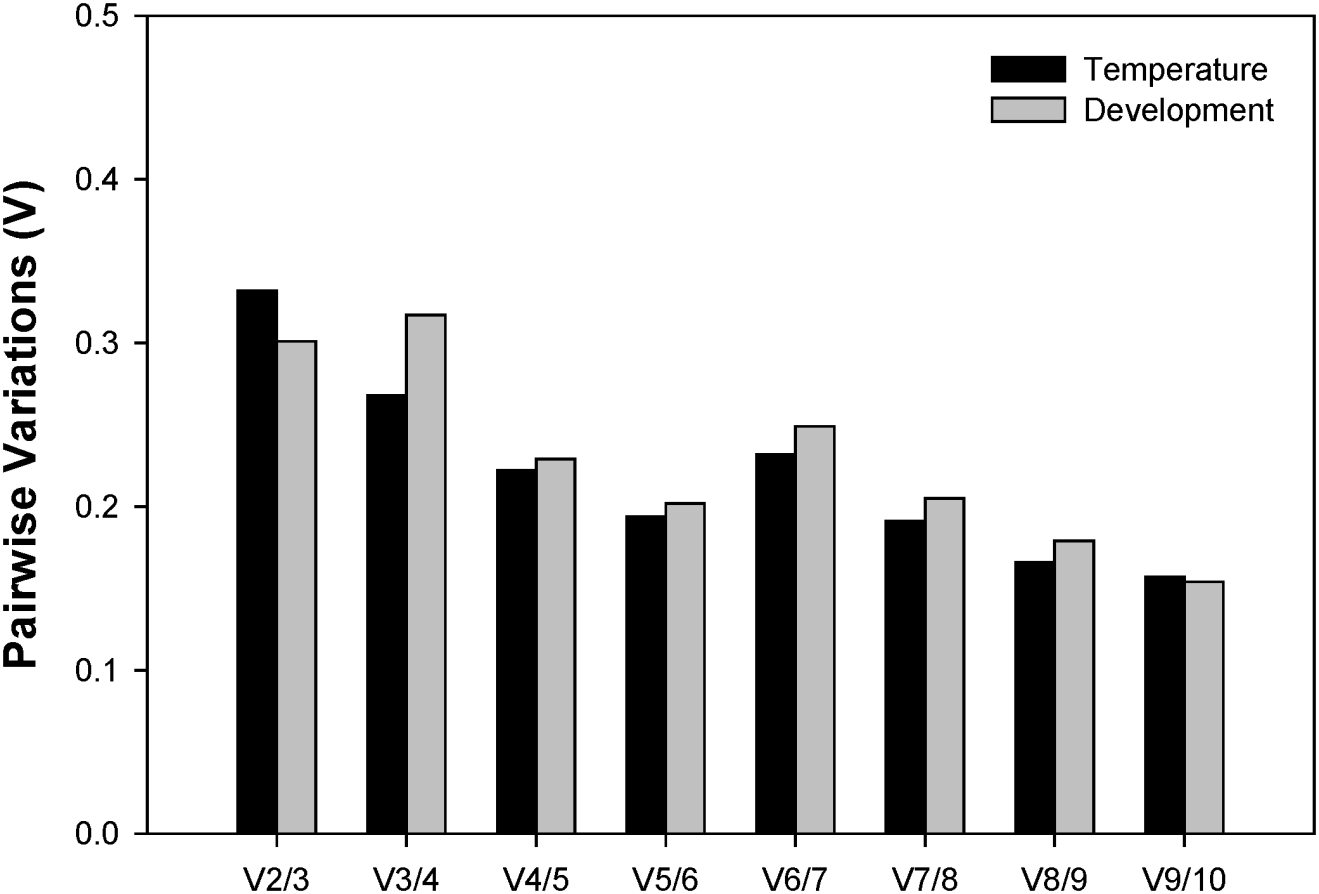
Pairwise variation (V) analysis of the candidate reference genes based on *geNorm*. The pair-wise variation (V_n_/V_n+1_) was analyzed to determine the best number of references genes demanded for qRT-PCR data normalization [1]. The value V6/7 is higher than V5/6; this is due to the inclusion of a relative unstable sixth gene. Increasing variation in this ratio corresponds to decreasing expression stability.

To decide the minimal number of genes mandatory for normalization, the V-value was computed by *geNorm*. *geNorm* analysis revealed that the pair-wise variation value V6/7 is higher than V5/6 (Fig 2). Increasing variation in this ratio corresponds to decreasing expression stability, due to the inclusion of a relatively unstable sixth gene. Therefore, five genes (*PRL14*, *RPS8*, *RPL11*, *ATPase*, and *RPS23*) are necessary for accurate normalization. Including a sixth reference gene has no significant effect on the normalization factor (Fig 2).

## Discussion

qRT-PCR quantification demands a comprehensive normalization by housekeeping genes to counteract confounding variations in experimental data. Housekeeping genes have been considered to be expressed in all cell types of the organism at a constant level to maintain basic cellular functions. However, there are no "universal" reference genes that are stably expressed and appropriate for the entire cell and tissue, and all kinds of test conditions [1]. Most gene expression studies in the literature use one single housekeeping gene; this will deeply influence the outcome of the statistical analysis and may bring about inaccurate data interpretation [29]. Therefore, customized reference gene selection under specific experimental conditions is highly recommended [11].

Recently, there is an influx of reference gene selection studies in insects, including convergent lady beetle, *Hippodamia convergens*; sweet potato whitefly, *Bemisia tabaci*; diamondback moth, *Plutella xylostella*; brown planthopper, *Nilaparvata lugens*; beet armyworm, *Spodoptera exigua*; oriental leafworm moth, *Spodoptera litura*; oriental fruit fly, *Bactrocera dorsalis*; Colorado potato beetle, *Leptinotarsa decemlineata*; soybean aphid, *Aphis glycines*; Russian wheat aphid, *Diuraphis noxia*; bird cherry-oat aphid, *Rhopalosiphum padi*; pea aphid, *Acyrthosiphon pisum*; bumblebees, *Bombus terrestris* and *Bombus lucorum*; western flower thrips, *Frankliniella occidentalis*; and honeybee, *Apis mellifera* [10–25]. Here, the expression profiles of ten HKGs from *A*. *craccivora* were evaluated across different developmental stages and temperature conditions. Our results are largely consistent with previous studies. For example, *RPS8* (the component of the *40S* ribosomal subunit) and *RPL14* (*60S* ribosomal subunit) were the most stable HKGs across different developmental stage and temperature conditions, whereas the expression of *18S* varied under the two conditions [14,16]. Not surprisingly, the comprehensive rankings (*RefFinder*) of these candidate reference genes under the two experimental conditions were, in principal, comparable to the rankings complied by the four algorithms, *geNorm*, *Normfinder*, *BestKeeper*, and the *ΔC*_*t*_ method, respectively (Tables 2 and 3). Based on the comprehensive analyses, *RPS8*, *RPL14*, and *RPL11* were the most stable *A*. *craccivora* HKGs under different developmental stages and temperature conditions.

There has been ongoing discussion about the optimal number of reference genes warrant for qRT-PCR analysis [9,14]. To prevent biased normalization, multiple instead of a single reference gene have been gradually adopted to normalize the expression of target genes under test conditions [30]. Our results showed that the pair-wise variation value of V6/7 is higher than that of V5/6 (Fig 2), suggesting that five reference genes are warranted for the accurate normalization in *A*. *craccivora* under different developmental stages and temperature conditions.

A phloem-feeding cowpea aphid, *A*. *craccivora*, is one of the key pests of cowpea, a major protein source for people in West Africa. Most recently, Roche 454-based pyrosequencing generated 176,262 raw reads from an *A*. *craccivora* transcriptome, and *de novo* assembly produced 7,647 transcripts [7]. Building on this newly developed genomic resource, we carried out the first reference gene selection study in one of the major pest species of cowpea. Although studies involving different developmental stages and /or temperature regimes have been limited [31–34], the advent of the Genomics Era will facilitate our understanding of *A*. *craccivora*, and eventually will lead to the development of integrated pest management strategies. Therefore, this study not only sheds light on establishing a standardized qRT-PCR procedure for quantification of gene expression in *A*. *craccivora*, but also lays a solid foundation for the genomics and functional genomics research in this sap-sucking insect pest.

## Supporting Information

S1 Fig The agrose gel electrophoresis of the ten candidate reference genes. M, EZ Load 100 bp Molecular Ruler; Templates in the PCR reactions were as follows: 1) *EF1A*; 2) *NADH*; 3) *HSP70*; 4) *18S*; 5)*12S*; 6) *RPS23*; 7) *RPS8*; 8) *RPL14*; 9) *RPL11*; 10) *ATPase*.
(TIFF)

S2 Fig Melting curves of ten candidate reference genes in *Aphis craccivora*. (TIFF)

S1 Table The mean and standard deviation (SD) of the *C*_*t*_ value for each candidate reference gene. (DOCX)

S2 Table Summary of mean and SD values of gene pairwise comparison using the *ΔC*_*t*_ method across different temperature. (DOCX)

S3 Table Summary of mean and SD values of gene pairwise comparison using the *ΔC*_*t*_ method under the developmental stage. (DOCX)

S4 Table Ranking of the candidate reference genes by *BestKeeper*. 18 samples were from temperature group as input.
(DOCX)

S5 Table Ranking of the candidate reference genes by *BestKeeper*. 12 samples were from developmental stage group as input.
(DOCX)
