# Supplementary figures and images for: Temperature and Development Impacts on Housekeeping Gene Expression in Cowpea Aphid, Aphis craccivora (Hemiptera: Aphidiae)
Source: PLoS One. 2015 Jun 19;10(6):e0130593. doi: 10.1371/journal.pone.0130593 (PMC4474611; doi:10.1371/journal.pone.0130593)

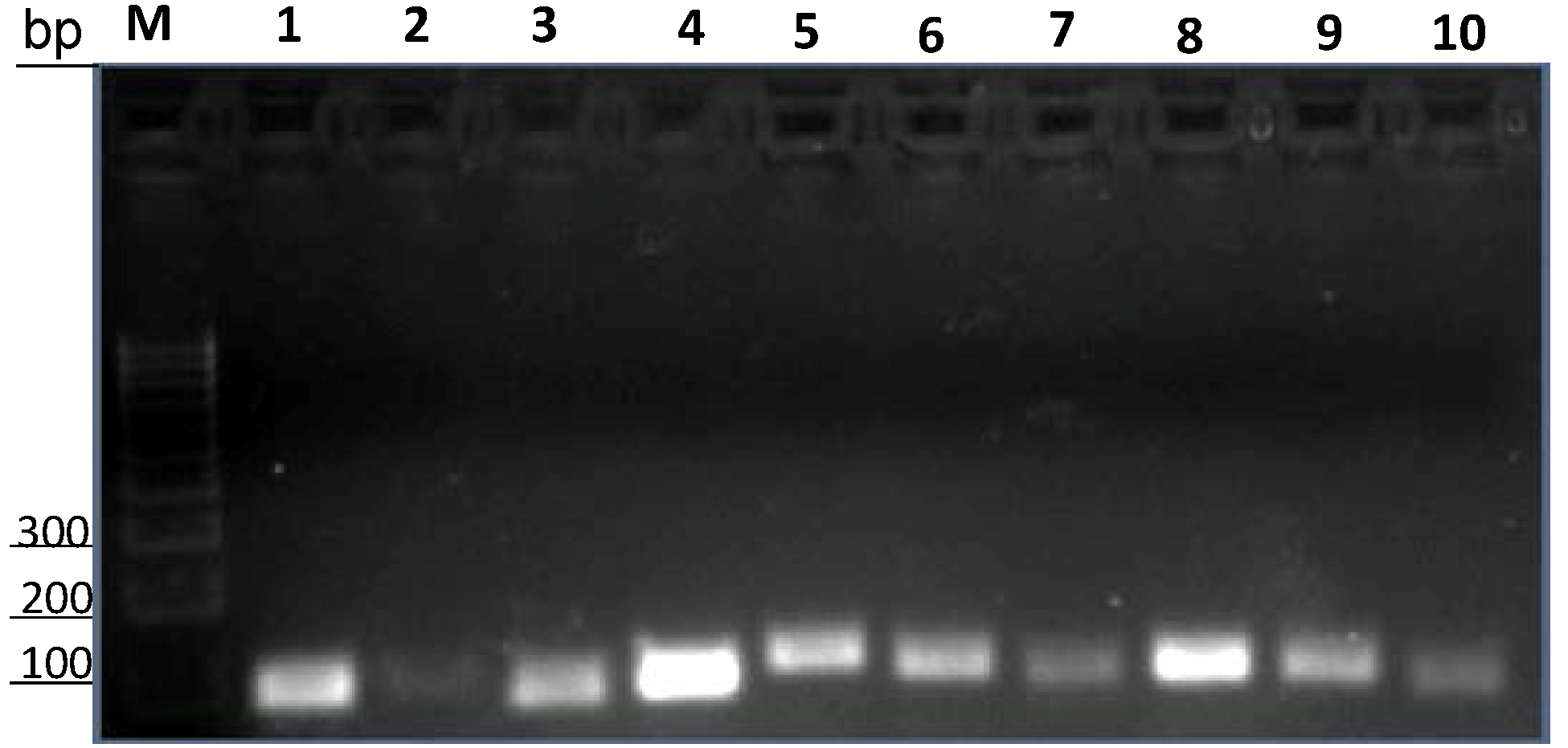

Supplement: S1 Fig — M, EZ Load 100 bp Molecular Ruler; Templates in the PCR reactions were as follows: 1) EF1A; 2) NADH; 3) HSP70; 4) 18S; 5)12S; 6) RPS23; 7) RPS8; 8) RPL14; 9) RPL11; 10) ATPase. (TIFF) [file pone.0130593.s001.tiff]

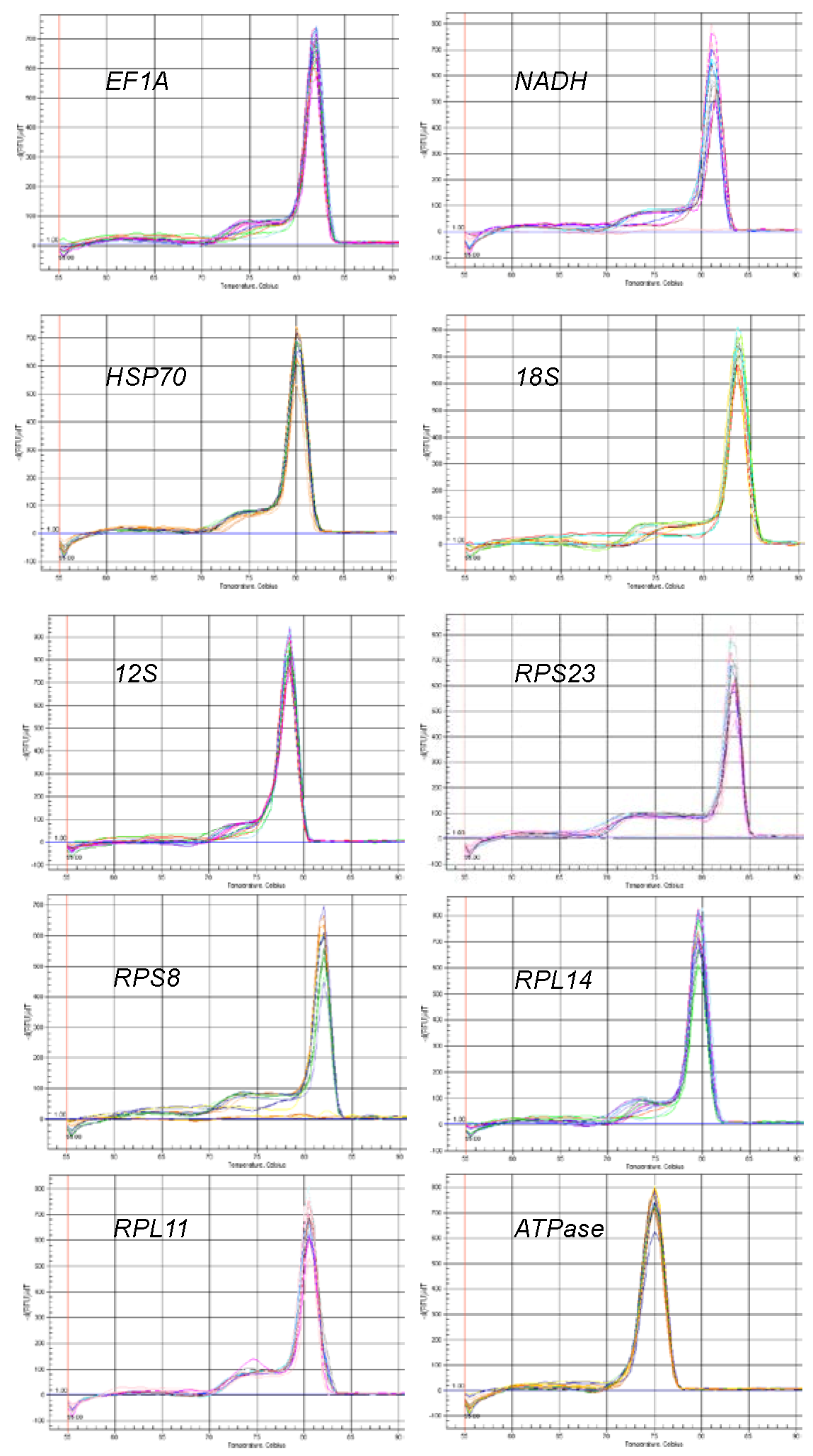

Supplement: S2 Fig — (TIFF) [file pone.0130593.s002.tiff]
