# Supplementary material for: Temperature and Development Impacts on Housekeeping Gene Expression in Cowpea Aphid, Aphis craccivora (Hemiptera: Aphidiae)
Source: PLoS One. 2015 Jun 19;10(6):e0130593. doi: 10.1371/journal.pone.0130593 (PMC4474611; doi:10.1371/journal.pone.0130593)
Supplement: S1 Table — (DOCX) [file pone.0130593.s003.docx]

**S1 Table. The mean and standard deviation (SD) of the *C_t_* value for each candidate reference gene**

| Genes | *C_t_* ±SD |
| --- | --- |
| *NADH* | 27.82±0.94 |
| *RPS8* | 25.53±0.69 |
| *ATPase* | 24.64±0.82 |
| *RPS23* | 22.23±0.65 |
| *RPL14* | 21.50±0.66 |
| *HSP70* | 21.38±0.94 |
| *RPL11* | 20.63±0.61 |
| *EF1A* | 20.46±1.09 |
| *12S* | 17.83±0.98 |
| *18S* | 8.50±0.77 |
