# Supplementary material for: Temperature and Development Impacts on Housekeeping Gene Expression in Cowpea Aphid, Aphis craccivora (Hemiptera: Aphidiae)
Source: PLoS One. 2015 Jun 19;10(6):e0130593. doi: 10.1371/journal.pone.0130593 (PMC4474611; doi:10.1371/journal.pone.0130593)
Supplement: S2 Table — (DOCX) [file pone.0130593.s004.docx]

**S2 Table. Summary of mean and SD values of gene pairwise comparison using the *ΔC_t_* method across different temperature**

| *Gene* |  | Pair 1 | Pair 2 | Pair 3 | Pair 4 | Pair 5 | Pair 6 | Pair 7 | Pair 8 | Pair 9 | Average SD |
| --- | --- | --- | --- | --- | --- | --- | --- | --- | --- | --- | --- |
| *EF1A* | Mean | 7.63 | 0.59 | -11.03 | -3.01 | 2.34 | 5.96 | 0.88 | 0.39 | 4.57 |  |
|  | SD | 1.44 | 1.02 | 2.06 | 1.90 | 2.18 | 1.86 | 2.04 | 1.88 | 1.94 | 1.81 |
| *NADH* | Mean | -7.63 | -7.05 | -18.67 | -10.64 | -5.30 | -1.67 | 6.76 | -7.25 | -3.07 |  |
|  | SD | 1.44 | 1.17 | 2.02 | 2.07 | 2.02 | 1.75 | 1.78 | 1.66 | 2.03 | 1.77 |
| *HSP70* | Mean | -0.59 | 7.05 | -11.62 | -3.60 | 1.75 | 5.38 | 0.29 | -0.20 | 3.98 |  |
|  | SD | 1.02 | 1.17 | 2.00 | 1.81 | 1.97 | 1.57 | 2.00 | 1.77 | 1.81 | 1.68 |
| *18S* | Mean | 11.03 | 18.67 | 11.62 | 8.02 | 13.37 | 17.00 | 11.91 | 11.42 | 15.60 |  |
|  | SD | 2.06 | 2.02 | 2.00 | 2.17 | 2.14 | 1.68 | 2.16 | 1.62 | 1.99 | 1.88 |
| *12S* | Mean | 3.01 | 10.64 | 3.60 | -8.02 | 5.34 | 8.97 | 3.88 | 3.39 | 7.58 |  |
|  | SD | 1.90 | 2.07 | 1.81 | 2.17 | 1.20 | 1.54 | 1.67 | 1.53 | 0.93 | 1.65 |
| *RPS23* | Mean | -2.34 | 5.30 | -1.75 | -13.37 | -5.34 | 3.63 | -1.46 | -1.95 | 2.23 |  |
|  | SD | 2.18 | 2.02 | 1.97 | 2.14 | 1.20 | 1.17 | 1.43 | 1.40 | 1.27 | 1.64 |
| *RPS8* | Mean | -5.96 | 1.67 | -5.38 | -17.00 | -8.97 | -3.63 | -5.09 | -5.58 | -1.40 |  |
|  | SD | 1.86 | 1.75 | 1.57 | 1.68 | 1.54 | 1.17 | 1.03 | 1.17 | 1.19 | 1.44 |
| *RPL14* | Mean | -0.88 | 6.76 | -0.29 | -11.91 | -3.88 | 1.46 | 5.09 | -0.49 | 3.69 |  |
|  | SD | 2.04 | 1.78 | 2.00 | 1.26 | 1.67 | 1.43 | 1.03 | 1.07 | 1.33 | 1.51 |
| *RPL11* | Mean | -0.39 | 7.25 | 0.20 | -11.42 | -3.39 | 1.95 | 5.58 | 0.49 | 4.18 |  |
|  | SD | 1.88 | 1.66 | 1.77 | 1.62 | 1.53 | 1.40 | 1.17 | 1.07 | 1.21 | 1.48 |
| *ATPase* | Mean | -4.57 | 3.07 | -3.98 | -15.60 | -7.58 | -2.23 | 1.40 | -3.69 | -4.18 |  |
|  | SD | 1.94 | 2.03 | 1.81 | 1.99 | 0.93 | 1.27 | 1.19 | 1.33 | 1.21 | 1.52 |
