# Supplementary material for: Temperature and Development Impacts on Housekeeping Gene Expression in Cowpea Aphid, Aphis craccivora (Hemiptera: Aphidiae)
Source: PLoS One. 2015 Jun 19;10(6):e0130593. doi: 10.1371/journal.pone.0130593 (PMC4474611; doi:10.1371/journal.pone.0130593)
Supplement: S3 Table — (DOCX) [file pone.0130593.s005.docx]

**S3 Table. Summary of mean and SD values of gene pairwise comparison using the *ΔC_t_* method under the developmental stage**

| *Gene* |  | Pair 1 | Pair 2 | Pair 3 | Pair 4 | Pair 5 | Pair 6 | Pair 7 | Pair 8 | Pair 9 | Average SD |
| --- | --- | --- | --- | --- | --- | --- | --- | --- | --- | --- | --- |
| *EF1A* | Mean | 7.88 | 0.93 | -11.10 | -2.97 | 2.27 | 6.06 | 0.80 | 0.45 | 4.60 |  |
|  | SD | 0.94 | 0.88 | 1.89 | 2.27 | 2.48 | 2.02 | 1.93 | 2.11 | 2.18 | 1.86 |
| *NADH* | Mean | -7.88 | -6.96 | -18.99 | -10.85 | -5.61 | -1.82 | -7.09 | -7.43 | -3.28 |  |
|  | SD | 0.90 | 0.96 | 1.80 | 2.22 | 2.19 | 1.72 | 1.66 | 1.71 | 2.09 | 1.69 |
| *HSP70* | Mean | -0.93 | 6.96 | -12.03 | -3.90 | 1.35 | 5.13 | -0.13 | -0.48 | 3.68 |  |
|  | SD | 0.88 | 1.01 | 1.78 | 2.10 | 2.24 | 1.68 | 1.86 | 1.85 | 1.86 | 1.69 |
| *18S* | Mean | 11.10 | 18.99 | 12.03 | 8.13 | 13.38 | 17.16 | 11.90 | 11.55 | 15.71 |  |
|  | SD | 1.89 | 1.88 | 1.78 | 2.29 | 2.33 | 1.63 | 1.44 | 1.56 | 2.20 | 1.89 |
| *12S* | Mean | 2.97 | 10.85 | 3.90 | -8.13 | 5.24 | 9.03 | 3.77 | 3.42 | 7.57 |  |
|  | SD | 2.27 | 2.31 | 2.10 | 2.29 | 1.33 | 1.61 | 1.61 | 1.74 | 0.72 | 1.78 |
| *RPS23* | Mean | -2.27 | 5.61 | -1.35 | -13.38 | -5.24 | 3.79 | -1.48 | -1.82 | 2.33 |  |
|  | SD | 2.48 | 2.29 | 2.24 | 2.33 | 1.33 | 1.25 | 1.45 | 1.42 | 1.25 | 1.78 |
| *RPS8* | Mean | -6.06 | 1.82 | -5.13 | -17.16 | -9.03 | -3.79 | -5.26 | -5.61 | -1.46 |  |
|  | SD | 2.02 | 1.80 | 1.68 | 1.63 | 1.61 | 1.25 | 0.78 | 1.07 | 1.26 | 1.45 |
| *RPL14* | Mean | -0.80 | 7.09 | 0.13 | -11.90 | -3.77 | 1.48 | 5.26 | -0.35 | 3.81 |  |
|  | SD | 1.93 | 1.73 | 1.86 | 1.44 | 1.61 | 1.45 | 0.78 | 0.89 | 1.44 | 1.46 |
| *RPL11* | Mean | -0.45 | 7.43 | 0.48 | -11.55 | -3.42 | 1.82 | 5.61 | 0.35 | 4.15 |  |
|  | SD | 2.11 | 1.78 | 1.85 | 1.56 | 1.74 | 1.42 | 1.07 | 0.89 | 1.44 | 1.54 |
| *ATPase* | Mean | -4.60 | 3.28 | -3.68 | -15.71 | -7.57 | -2.33 | 1.46 | -3.81 | -4.15 |  |
|  | SD | 2.18 | 2.18 | 1.86 | 2.20 | 0.72 | 1.25 | 1.26 | 1.44 | 1.44 | 1.62 |
