# Supplementary material for: Temperature and Development Impacts on Housekeeping Gene Expression in Cowpea Aphid, Aphis craccivora (Hemiptera: Aphidiae)
Source: PLoS One. 2015 Jun 19;10(6):e0130593. doi: 10.1371/journal.pone.0130593 (PMC4474611; doi:10.1371/journal.pone.0130593)
Supplement: S4 Table — 18 samples were from temperature group as input. (DOCX) [file pone.0130593.s006.docx]

**S4 Table. Ranking of the candidate reference genes by *BestKeeper**** **.** 18 samples were from temperature group as input.

| Gene | GM  [C_t_] | AM | Min | Max | SD | CV | [r] | p-value | Ranking | |
| --- | --- | --- | --- | --- | --- | --- | --- | --- | --- | --- |
|  |  | [C_t_] | [C_t_] | [C_t_] | [±C_t_] | [% C_t_] |  |  | [r] | SD |
| *EF1A* | 20.41 | 20.46 | 17.8 | 22.72 | 1.30 | 6.34 | 0.477 | 0.045 | *RPL11* | *RPL14* |
| *NADH* | 28.07 | 28.1 | 25.98 | 30.49 | 1.14 | 4.04 | 0.249 | 0.319 | *RPS8* | *ATPase* |
| *HSP70* | 21.01 | 21.05 | 18.04 | 22.96 | 1.01 | 4.82 | 0.402 | 0.099 | *RPL14* | *RPL11* |
| *18S* | 9.33 | 9.43 | 7.23 | 11.91 | 1.16 | 12.33 | 0.462 | 0.053 | *EF1A* | *RPS8* |
| *12S* | 17.41 | 17.45 | 15.29 | 19.58 | 1.04 | 5.97 | 0.359 | 0.143 | *18S* | *RPS23* |
| *RPS23* | 22.77 | 22.8 | 20.9 | 24.86 | 1.00 | 4.39 | 0.177 | 0.482 | *HSP70* | *HSP70* |
| *RPS8* | 26.41 | 26.43 | 24.64 | 28.00 | 0.94 | 3.55 | 0.624 | 0.006 | *ATPase* | *12S* |
| *RPL14* | 21.31 | 21.34 | 19.06 | 23.12 | 0.86 | 4.05 | 0.605 | 0.008 | *12S* | *NADH* |
| *RPL11* | 20.82 | 20.85 | 18.94 | 23.48 | 0.90 | 4.33 | 0.692 | 0.001 | *NADH* | *18S* |
| *ATPase* | 25.01 | 25.03 | 23.01 | 27.16 | 0.89 | 3.54 | 0.375 | 0.125 | *RPS23* | *EF1A* |

"*": Two criteria are considered: Pearson’s correlation coefficient and *BestKeeper* computed SD values. The stability of a gene is directly proportional to the [r] value, while it is inversely proportional to the SD value. GM: the geometric mean of Ct; AM: the arithmetic mean of Ct; Min and Max: the extreme values of Ct, respectively; SD: the standard deviation of Ct; CV: the coefficient of variance expressed as a percentage on Ct level; r: Pearson’s correlation coefficient.
