# Supplementary material for: Temperature and Development Impacts on Housekeeping Gene Expression in Cowpea Aphid, Aphis craccivora (Hemiptera: Aphidiae)
Source: PLoS One. 2015 Jun 19;10(6):e0130593. doi: 10.1371/journal.pone.0130593 (PMC4474611; doi:10.1371/journal.pone.0130593)
Supplement: S5 Table — 12 samples were from developmental stage group as input. (DOCX) [file pone.0130593.s007.docx]

**S5 Table. Ranking of the candidate reference genes by *BestKeeper**** 12 samples were from developmental stage group as input.

| Gene | GM  [C_t_] | AM | Min | Max | SD | CV | [r] | p-value | Ranking | |
| --- | --- | --- | --- | --- | --- | --- | --- | --- | --- | --- |
|  |  | [C_t_] | [C_t_] | [C_t_] | [±C_t_] | [% C_t_] |  |  | [r] | SD |
| *EF1A* | 20.31 | 20.36 | 17.98 | 22.39 | 1.26 | 6.18 | 0.299 | 0.346 | *RPL14* | *HSP70* |
| *NADH* | 28.22 | 28.24 | 26.45 | 30.49 | 1.05 | 3.72 | 0.231 | 0.47 | *RPL11* | *RPS8* |
| *HSP70* | 21.26 | 21.29 | 18.93 | 22.96 | 0.88 | 4.15 | 0.276 | 0.385 | *18S* | *RPL11* |
| *18S* | 9.14 | 9.26 | 7.23 | 11.91 | 1.40 | 15.07 | 0.700 | 0.011 | *RPS8* | *RPL14* |
| *12S* | 17.35 | 17.39 | 15.29 | 19.58 | 1.04 | 6.01 | 0.183 | 0.568 | *ATPase* | *ATPase* |
| *RPS23* | 22.6 | 22.63 | 20.9 | 24.86 | 1.04 | 4.57 | 0.001 | 0.997 | *EF1A* | *RPS23* |
| *RPS8* | 26.4 | 26.42 | 24.64 | 27.71 | 0.95 | 3.60 | 0.670 | 0.017 | *HSP70* | *12S* |
| *RPL14* | 21.12 | 21.16 | 19.06 | 23.12 | 1.01 | 4.77 | 0.860 | 0.001 | *NADH* | *NADH* |
| *RPL11* | 20.78 | 20.81 | 18.94 | 23.48 | 0.97 | 4.67 | 0.748 | 0.005 | *12S* | *EF1A* |
| *ATPase* | 24.94 | 24.96 | 23.01 | 27.16 | 1.03 | 4.13 | 0.376 | 0.228 | *RPS23* | *18S* |

"*": Two criteria are considered: Pearson’s correlation coefficient and *BestKeeper* computed SD values. The stability of a gene is directly proportional to the [r] value, while it is inversely proportional to the SD value. GM: the geometric mean of Ct; AM: the arithmetic mean of Ct; Min and Max: the extreme values of Ct, respectively; SD: the standard deviation of Ct; CV: the coefficient of variance expressed as a percentage on Ct level; r: Pearson’s correlation coefficient.
